# Perioperative infections as a prognostic risk factor in hepatocellular carcinoma and cholangiocellular carcinoma: a comparative analysis

**DOI:** 10.1186/s12957-024-03651-8

**Published:** 2025-01-07

**Authors:** Markus S. Jördens, Hannah C. Oswald, Lisa Heinrichs, Nathalie Gassmann, Linda Wittig, Tom Luedde, Sven H. Loosen, Christoph Roderburg, Wolfram T. Knoefel, Georg Fluegen

**Affiliations:** 1 Department for Gastroenterology, Hepatology and Infectious Diseases, Medical Faculty, Heinrich Heine University Düsseldorf, University Hospital Düsseldorf, Moorenstr. 5, Düsseldorf, 40225 Germany; 2 Department for General, Visceral and Pediatric Surgery, Medical Faculty, Heinrich Heine University Düsseldorf, University Hospital Düsseldorf, Düsseldorf, Germany

**Keywords:** HCC, CCA, Perioperative infections, Survival, Prognostic marker

## Abstract

**Background:**

Hepatocellular Carcinoma (HCC) and cholangiocellular adenocarcinoma (CCA) are the most common primary liver tumors representing a major global health burden. In early disease stages, tumor resection may provide long-term survival in selected patients. However, morbidity and mortality rates are still relatively high after extended liver surgery with perioperative bacterial infections representing major complications. In this study, we evaluate the impact of perioperative infection on the postoperative overall survival (OS) of patients undergoing resection of HCC or CCA.

**Material and methods:**

Two hundred two patients that received liver surgery for HCC (139) or CCA (63) at our tertiary referral center were included between 2008 and 2020. Infection prior or after surgery was assessed using patient documentation and correlated to patients´ survival rates and other clinical characteristics.

**Results:**

Patients with perioperative infection displayed a significantly impaired OS compared to patients without a documented infection (419 (95% CI: 262–576) days vs. 959 (95% CI: 637–1281) days; log rank X^2^(1) = 10.28; *p* < 0.001). Subgroup analysis revealed that this effect was only observed among HCC patients, while the outcome of CCA patients was independent of pre- or postoperative infections. Moreover, non-anatomical resection of liver tumors was beneficial in patients with HCC (1541 (95%CI: 1110–1972) vs. 749 (95%CI: 0–1528) days; log rank X^2^(1) = 5.387; *p* = 0.02) but not CCA.

**Conclusion:**

Perioperative infection is an important prognostic factor after surgery for HCC but not CCA.

**Supplementary Information:**

The online version contains supplementary material available at 10.1186/s12957-024-03651-8.

## Introduction

Hepatocellular carcinoma (HCC) and cholangiocellular carcinoma (CCC) represent two distinct entities within the spectrum of primary liver malignancies, each characterized by unique etiological factors, clinical presentations, and prognostic outcomes [1, 2]. Despite recent advances in diagnostic modalities and multimodal therapeutic interventions, both HCC and CCC remain formidable challenges in oncology, with limited effective treatment options and relatively poor overall survival rates [3–6].

The perioperative period, encompassing the time from some days before surgical resection to postoperative recovery, plays a critical role in shaping the trajectory of patient outcomes in liver cancer. Perioperative complications, including infections, have emerged as significant determinants of postoperative morbidity and mortality in various surgical contexts. However, the impact of perioperative infections on the prognosis of patients undergoing surgical management for HCC and CCC remains incompletely understood [7–9].

In recent years, accumulating evidence has suggested a potential association between perioperative infections and adverse outcomes in patients with hepatocellular carcinoma [10]. Studies have implicated infections in exacerbating systemic inflammation, compromising immune function, and predisposing patients to postoperative complications, thereby influencing long-term survival outcomes [11]. Conversely, the prognostic significance of perioperative infections in cholangiocellular carcinoma remains less elucidated, with limited data available to inform clinical practice and decision-making.

In this context, our study seeks to investigate the differential impact of perioperative infections on the prognosis of patients undergoing surgical resection for hepatocellular carcinoma and cholangiocellular carcinoma. By conducting a comparative analysis of clinical outcomes, including postoperative morbidity, mortality, and long-term survival, we aim to elucidate whether perioperative infections serve as a prognostic risk factor in primary liver cancer.

## Material and methods

### Patient cohort

**Fig. 1:**
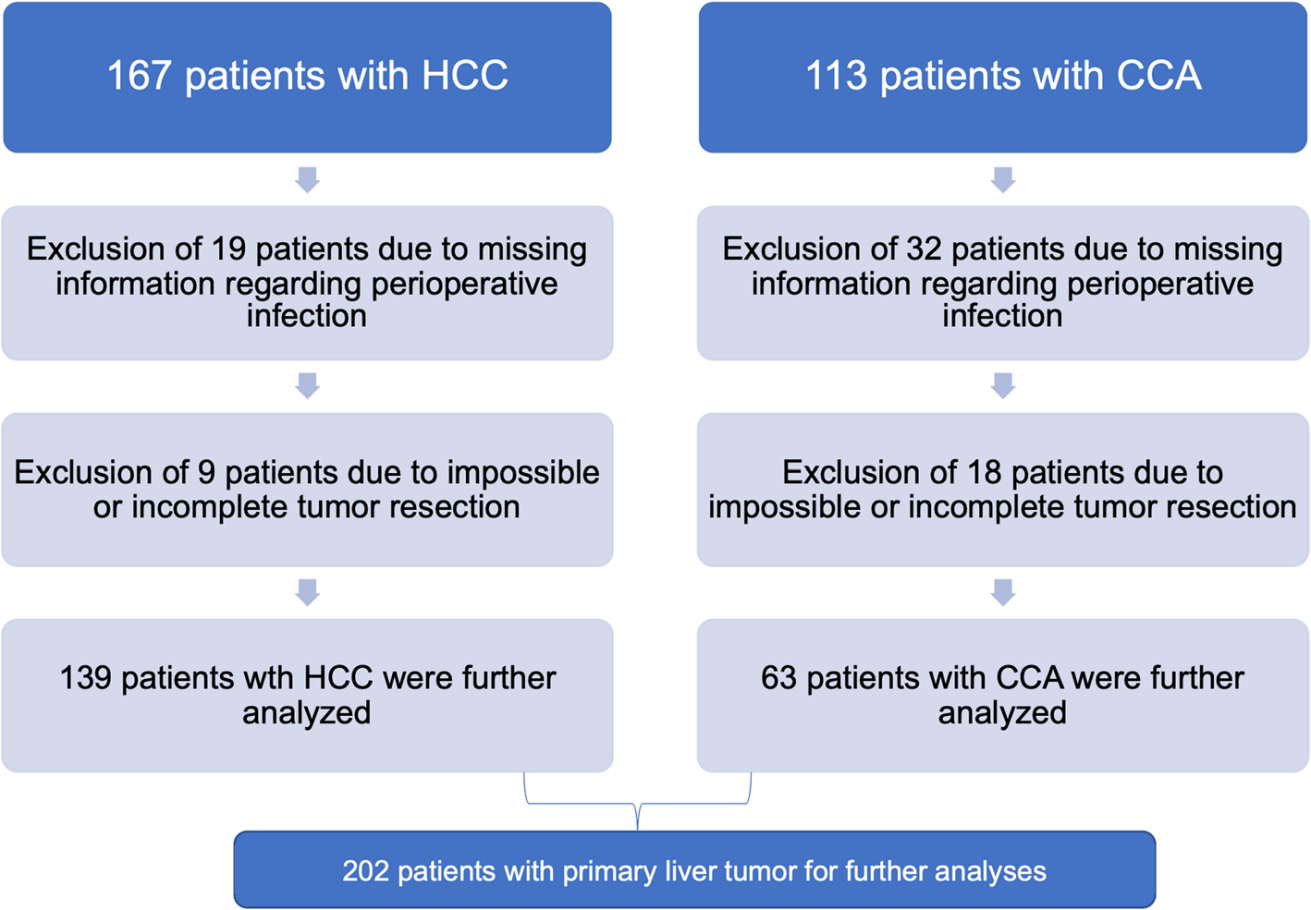
Patient selection

For our analyses, we included patients undergoing surgery for hepatocellular carcinoma (HCC) or cholangiocellular adenocarcinoma (CCA) at the Department of General, Visceral and Pediatric Surgery, University Hospital Düsseldorf, Germany, between 2008 and 2020. 167 patients with HCC and 81 patients with CCC were identified. 19 HCC patients and 32 CCA patients had to be excluded from further analyses due to lack of information regarding infection, further 9 patients with HCC and 18 with CCA had to be excluded as they didn’t receive complete tumor resection (Fig. 1). The study was approved by the ethics committee of the medical faculty, Heinrich Heine university Düsseldorf (2021–1418).

### Patient parameters

Laboratory values and parameters used for the statistical analyses were obtained from the available patient files and the clinic's patient documentation system. Perioperative infection was defined as the detection of a pathogen in bile or blood, as well as the documentation of an infection in the physician's note and perioperatively elevated inflammation values with pathogen detection prior, during or within 14 days after operation with direct relation to the operation. Serum markers CRP, leucocyte count and PCT were used to define infection.

### Statistical analysis

All statistical analysis was performed using SPSS 27 (SPSS, Chicago, IL, USA). *P*-values < 0.05 were considered statistically significant, Hazard ration (HR) and 95% confidence intervals (95% CI) are displayed. Kaplan–Meier curve analysis was used to investigate the impact of patients` characteristics on overall survival, Log-rank test to compare groups.

The prognostic impact of different variables regarding overall survival was estimated using univariate and multivariate Cox-regression analyses.

## Results

### Patient characteristics

202 patients with either HCC (139) or CCA (63) were included into analysis. 60 patients (29.7%) were female and 142 were male (70.3%). The mean age was 66.7 years, and the mean BMI was 27.1 kg/m^2^. 63 (31.2%) patients underwent hemihepatectomy and 138 (68.3%) non-anatomical resection. Median overall survival for the entire cohort was 503 days, for HCC 529.5 days, and for CCA 290 days. Among patients with CCA, 20 (31.7%) patients received adjuvant chemotherapy. Among patients with HCC, 40.3% had any kind of perioperative infection, and among patients with CCA, it was 44.4%. For additional parameters, see Table 1.

**Table 1:** Patient Cohort

Parameter	Patient cohort
HCC + CCA patients	*n* = 202
sex (%, n):	
male	70.3 (142)
female	29.7 (60)
age (years; mean/median)	66.96/69.00
BMI groups (kg/m^2^, %. n):	
BMI < 24.9	34.2 (69)
BMI 24.9–29.9	36.1 (73)
BMI > 29.9	26.7 (96)
infection (%, n):	
yes	41.6 (84)
perioperative	10.4 (21)
only postoperative	31.2 (63)
no	58.4 (118)
Cirrhosis (%, n)	
All	57.4 (116)
HCC	73.4 (102)
CCA	22.2 (14)
Concomitant Conditions (%, n):	
Diabetes mellitus type 1 or type 2	35.6 (72)
Hypertension	50.5 (102)
Hepatitis B	23.8 (48)
Hepatitis C	24.3 (49)
Progressive disease (%, n):	
yes	41.6 (84)
no	46.0 (93)
Overall survival (days; median)	503.00

*BMI* Body mass index, *CCA* Cholangiocellular adenocarcinoma, *HCC* hepatocellular carcinoma; perioperative: Documented infection before or within 14 days after operation; only postoperative: Documented infection only after operation

### Perioperative infection is a prognostic marker for overall survival after resection of primary liver tumors

**Fig. 2:**
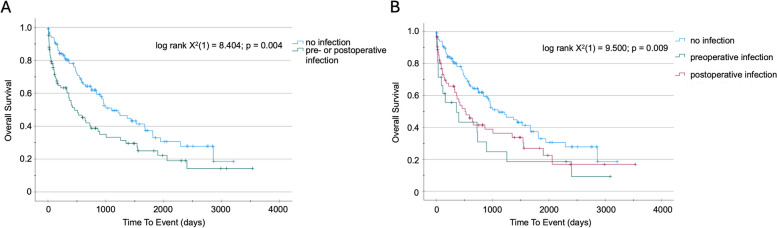
Perioperative infection is a prognostic factor for overal survival after surgery for primary liver tumors. **A** Kaplan–Meier survival analysis of patients with or without infection receiving surgery for primary liver tumors. **B** Kaplan–Meier survival analysis of patients receiving surgery for primary liver tumors with preoperative, postoperative or no infection

Overall survival (OS) of patients with or without any perioperative infection after resection of a primary liver tumor (HCC or CCA) was compared using Kaplan–Meier analysis. Perioperative infections were defined as follows: i) Detection of pathogens in blood, bile or intra-operation swaps in combination with elevated laboratory markers for inflammation (CRP, leucocyte count); ii) elevated laboratory markers of inflammation in combination with reported infection in the patients chart or doctors letter. Patients with any perioperative infection had a significantly worse OS compared to patients without perioperative infection (459 (95% CI: 202–716) days vs. 1099 (95% CI: 680–1518) days; log rank X^2^(1) = 8.40; *p* = 0.004; Fig. 2A). When comparing the timing of perioperative infections, patients with infections prior to the operation seem to have even worse OS than patients with postoperative infections, although statistical significance was only reached when comparing the group of patients with no infections to the others. (preoperative infection: 360 (95% CI 0–797) days vs. postoperative infection: 512 (95% CI 249–775) days vs. no infection: 1099 (95% CI 680–1518) days; log rank X^2^(2) = 9.500; *p* = 0.009; Fig. 2B). Our findings were corroborated using Cox regression analysis, showing that perioperative infection was a relevant negative predictor for OS in patients operated for a primary liver tumor (HR 1.709 (95%CI: 1.184–2.467); p = 0.004; Table 2). Univariate cox regression analysis further identified preoperatively elevated inflammation markers (CRP: HR 1.171 (95%CI: 1.112–1.233); *p* < 0.001), leucocyte count (HR 1.092 (95%CI: 1.022–1.166); p = 0.009) or the coagulation marker PTT (1.030 (95%CI: 1.013–1.047); *p* < 0.001) as predictors for worse OS in this patient group.

**Table 2:** Univariate Cox regression analysis for OS in patients operated on HCC or CCA

Parameter	*p*-value	Hazard Ratio (95% CI)	n (202)
Age	0.519	1.005 (0.988–1.025)	202
Sex	0.532	1.139 (0.757–1.714)	202
Weight	0.127	0.992 (0.982–1.002)	196
Hight	0.996	1.000 (0.980–1.020)	196
BMI	0.059	0.962 (0.924–1.001)	196
Infection	0.004	1.709 (1.184–2.467)	202
Active viral hepatitis	0.036	0.636 (0.417–0972)	200
Non-anatomical (limited) liver resection	0.097	0.722 (0.492–1.060)	202
Sodium	0.120	0.956 (0.904–1.012)	198
Potassium	0.646	1.011 (0.964–1.061)	197
Calcium	0.001	0.180 (0.063–0.513)	195
Creatinin	0.696	1.051 (0.819–1.349)	198
GFR	0.897	0.999 (0.992–1.007)	196
Urea	0.603	1.003 (0.992–1.014)	197
Uric acid	0.697	1.008 (0.967–1.052)	63
Bilirubin	0.166	1.035 (0.986–1.088)	183
CRP	< 0.001	1.171 (1.112–1.233)	196
LDH	0.035	1.001 (1.000–1.001)	86
AST	0.038	1.003 (1.000–1.006)	196
γGT	< 0.001	1.001 (1.001–1.002)	193
AP	< 0.001	1.002 (1.001–1.002)	149
Albumin	0.922	0.998 (0.968–1.029)	51
TSH	0.163	1.092 (0.965–1.236)	158
leucocyte count	0.009	1.092 (1.022–1.166)	197
Hb	0.894	1.001 (0.988–1.014)	198
Thrombocytes	0.295	1.001 (0.999–1.003)	198
CEA	0.096	1.069 (0.988–1.157)	51
AFP	0.057	1.000 (1.000–1.000)	65
CA-19–9	0.047	1.000 (1.000–1.000)	60
INR	0.063	4.440 (0.924–21.345)	185
PTT	< 0.001	1.030 (1.013–1.047)	185

*BMI* Body-Mass Index, *GFR* glomerular filtration rate, *CRP* C-reactive protein, *LDH* lactate dehydrogenase, *AST* aspartate-aminotransferase, *γGT* γ-glutamyltransferase, *AP* Alkaline phosphatase, *TSH* Thyroid-stimulating hormone, *Hb* Hemoglobin, *CEA* Carcinoembryonic antigen, *AFP* α-fetoprotein *CA19-9* Carbohydrate antigen 19–9, *INR* International normalized ratio, *aPTT* activated partial thromboplastin time

#### Patients with active viral hepatitis have better overall survival following primary liver tumor resection

**Fig. 3:**
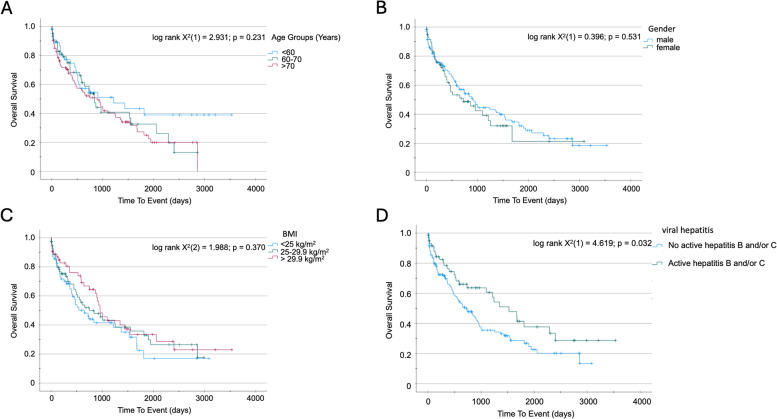
Active (detectable virus load/untreated) viral hepatitis B or C influences overall survival after surgery for primary liver tumors. Kaplan–Meier survival analysis for influence of age group (**A**), gender (**B**), BMI group (**C**) or active viral hepatitis **B** or **C** (**D**) on overall survival after surgery for primary liver tumors

To identify further relevant factors for postoperative survival in our cohort, we used Kaplan–Meier curve analysis. We did not detect a significant difference in the survival regarding patient age group (< 60 years: 1217 (95% CI 194–2240) days v. 60–70 years: 841 (95% CI 677–1005) days vs. > 70 years: 893 (95% CI 530–1256) days; log rank X^2^(2) = 2.931; *p* = 0.231; Fig. 3A). Furthermore, neither sex (female 724 (95% CI 208–1240) days vs. male 907 (95% CI 691–1123) days; log rank X^2^(1) = 0.392; *p* = 0.531; Fig. 3B) nor BMI (< 24.9 kg/m^2^: 649 (95% CI 360–938) days vs. 24.9–29.9 kg/m^2^: 749 (95% CI 305–1193) days vs. > 29.9 kg/m^2^: 959 (95% CI 741–1177) days; log rank X^2^(2) = 1.988; *p* = 0.370; Fig. 3C) had a significant impact on postoperative survival in our cohort. Interestingly, patients with liver disease related to chronic viral hepatitis had a clear trend towards better OS after resection of primary liver tumors, compared to the non-viral HCC/CCA patients. Further analysis showed a significant effect only for still active viral hepatitis (active/chronic hepatitis B/C (1526 (95% CI 969–2083) days vs. no active/chronic hepatitis B/C 724 (95% CI 482–966) days; log rank X^2^(1) = 4.454; *p* = 0.035; Fig. 3D). Moreover, univariate Cox regression analysis revealed viral infection as predictive marker for OS after primary liver tumor resection (HR 0.636 (0.417–0.972); p = 0.036, Table 2).

### Patients with HCC but not CCA benefit from non-anatomical liver resection

**Fig. 4:**
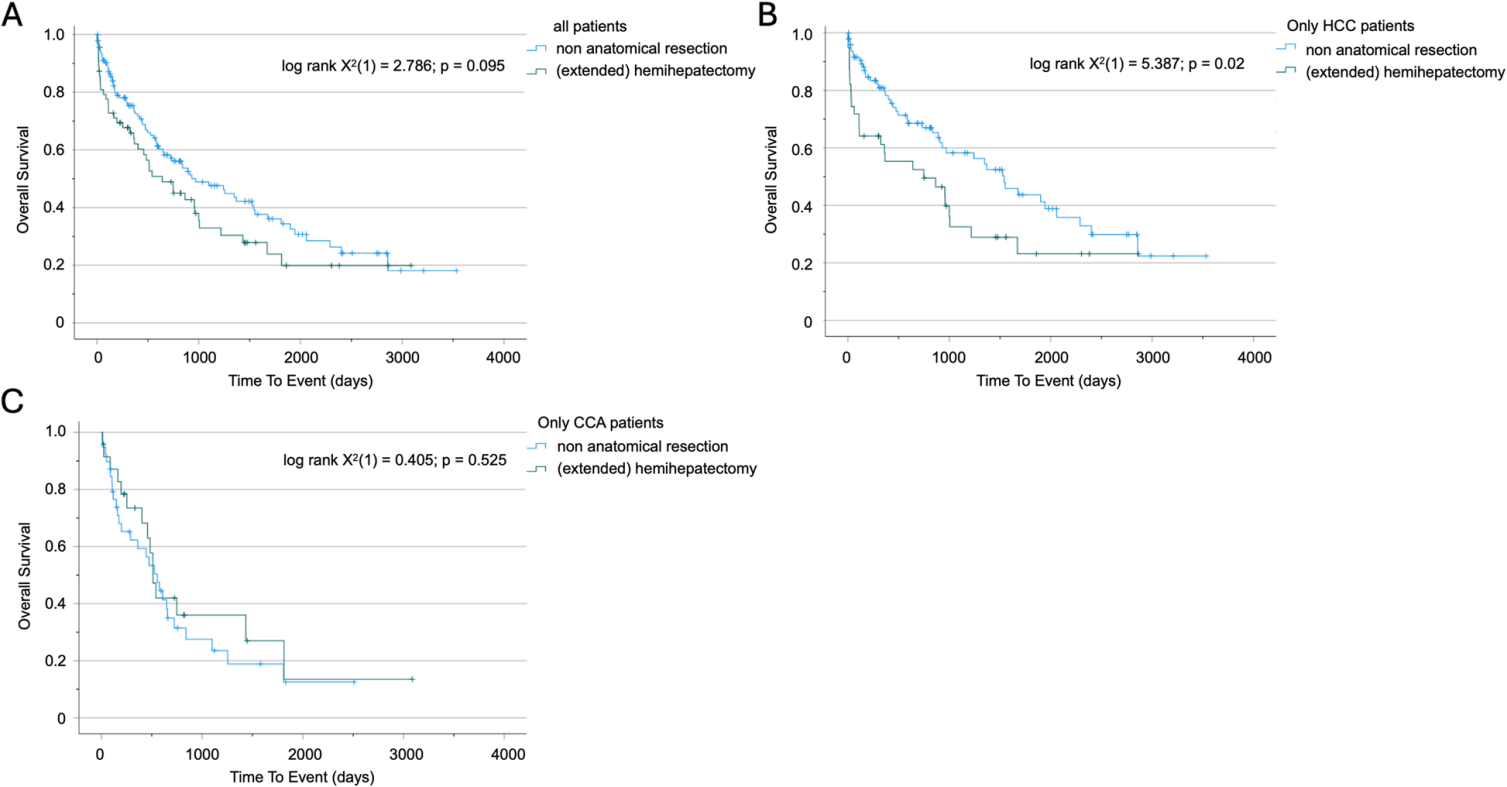
Extent of surgery impacts overall survival in patients with HCC but not CCA. **A** Kaplan–Meier survival analysis of patients receiving either non anatomical resection or (extended) hemihepatectomy for primary liver cancer. **B** Kaplan–Meier survival analysis of patients receiving either non anatomical resection or (extended) hemihepatectomy for HCC. **C** Kaplan–Meier survival analysis of patients receiving either non anatomical resection or (extended) hemihepatectomy for CCA

Since the extend of surgical resection can differ significantly depending of the size and location of the lesion, we investigated if resection method and extend of resection had an influence on postoperative survival in our cohort. As expected, patients undergoing a non-anatomical and somewhat limited resection of primary liver tumors had a clear trend towards improved OS compared to patients undergoing hemihepatectomy or anatomical hepatectomies (969 (95%CI: 521–1417) vs. 642 (95%CI: 280–1004) days; log rank X^2^(1) = 2.786; *p* = 0.095; Fig. 4A). Strikingly, when looking at HCC and CCA separately, the beneficial effect on OS of limited liver tissue resection remained only the case of patients with HCC (1541 (95%CI: 1110–1972) vs. 749 (95%CI: 0–1528) days; log rank X^2^(1) = 5.387; *p* = 0.02; Fig. 4B) but not in patients with CCA (555 (95%CI: 376–734) vs. 512 (95%CI: 431–593) days; log rank X^2^(1) = 0.405; *p* = 0.525; Fig. 4C). These results were confirmed in univariate Cox regression analysis, indicating that non-anatomical (limited) liver resection has a beneficial influence on OS just in patients suffering from HCC (HR 0.567 (0.349–0.921); *p* = 0.022; Supplemental Table 1).

### Perioperative infection is a relevant prognostic marker for patients operated with HCC but not CCA

**Fig. 5:**
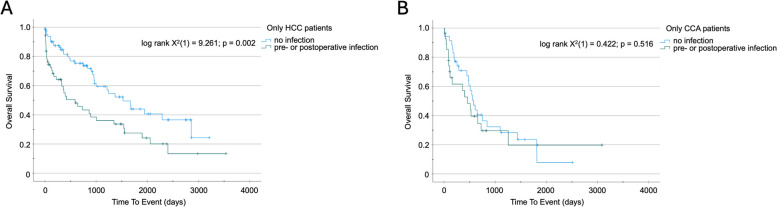
Perioperative infection is a prognostic factor for overall survival for patients with HCC but not CCA after tumor resection. **A** Kaplan–Meier survival analysis of patients with or without perioperative infection receiving surgery for HCC. **B** Kaplan–Meier survival analysis of patients with or without perioperative infection receiving surgery for CCA

To further evaluate the value of perioperative infection as a prognostic marker for OS in patients with primary liver tumors, we compared the HCC and CCA sub-cohorts separately. Patients receiving resection due to HCC showed a significantly worse OS if they had a perioperative infection, compared to those without infection (590 (95% CI: 147–1033) vs. 1526 (95% CI: 1038–2014) days; log rank X^2^(1) = 9.261; p 0.002; Fig. 5A). Surprisingly, we could not identify a similar difference in OS for CCA patients with or without perioperative infection (459 (95% CI: 230–688) vs. 575 (449–701) days; log rank X^2^(1) = 0.422; *p* = 0.516; Fig. 5B). This finding was again corroborated by Cox regression analysis, indicating that perioperative infection was indeed a relevant positive predictor in surgical patients suffering from HCC (HR 2.024 (1.273–3.217); *p* = 0.003; suppl. Table 1) but not CCA (HR 1.223 (0.665- 2.249); *p* = 0.517; suppl. Table 2)).

### Multivariate cox regression analysis

To identify independent prognostic factors for survival after surgery for HCC or CCA we performed multivariate Cox regression analysis including parameters we could obtain from at least 150 patients and that had shown statistical significance in univariate Cox regression analysis (*p* < 0.05). As infection and elevated inflammation markers like CRP or leucocyte count usually correlate with each other, in these analyses, none of the selected factors was statistically independently relevant for survival after surgery (Table 3).

**Table 3:** Multivariate Cox regression analysis for patients operated on a primary liver tumor (HCC and CCA)

Parameter	*p*-value	Hazard Ratio (95% CI)
Infection	0.522	1.155 (0.743–1.793)
Calcium	0.372	0.557 (0.154–2.011)
CRP	0.066	1.112 (0.993–1.246)
AST	0.491	1.001 (0.998–1.005)
γGT	0.068	1.001 (1.000–1.002)
leucocyte count	0.896	0.993 (0.895–1.102)
aPTT	0.093	1.017 (0.887–1.037)

*CRP* C-reactive protein, *AST* aspartate-aminotransferase, *γGT* γ-glutamyltransferase, *aPTT* activated partial thromboplastin time. For multivariate Cox regression analysis values from univariate Cox regression analysis with *p* < 0.05 and n ≥ 150 were included. *p*-value < 0.05 is statistically significant

## Discussion

In this retrospective study, we analysed the impact of perioperative infections on overall survival in 202 patients who underwent liver surgery for primary liver cancer (HCC and CCA). Patients with perioperative infection had significantly worse overall survival compared with patients without clinically apparent infection. Strikingly, subgroup analyses showed that this observation was only true for patients with HCC, but not for those with CCA. Furthermore, patients with HCC who underwent limited liver resection had a better OS compared to patients who underwent hemihepatectomy.

Liver cirrhosis represents the most important risk factor for HCC but has only a relatively weak association with the development of CCA. In line, 73.4% of our patients with HCC had concomitant liver cirrhosis, while only 22.2% of CCA developed within a cirrhotic liver. Since an impaired liver function represent a major risk factor for an unfavorable clinical course in patients with infectious diseases, we hypothesized that the different effect of perioperative overall survival in patients with HCC and CCA might partially be due to the different frequencies of liver cirrhosis in the different sub-cohorts (HCC or CCA) of this study [12]. Interestingly, Kaplan–Meier curve analyses revealed that the main difference in survival occurs within the first weeks after surgery, while at later time points, the curves run parallel to each other, arguing for the need of optimal patient selection and preparation before surgery. We therefor hypothesize that any perioperative infections are an important predictor for short term postoperative survival especially in patients with HCC. Whether further immunological factors triggered by the perioperative infection might play a role in determining the patients´ survival in the long-run cannot be determined by our retrospective data analysis.

The imminent danger of infections in patients with liver cirrhosis is well studied [12]. Patients with cirrhosis are at increased risk for bacterial infections and the mortality of these patients is increased fourfold compared to patients without cirrhosis [13–15]. In cirrhotic patients, the immune system is deficient, but constantly active at the same time [12]. Both innate and adaptive immune response are affected, including monocytes, T-cells and B-cell, providing a mechanistic base for the effects observed in our analyses [16, 17]. As a consequence, our data stress for a strict surveillance of infections in cirrhotic patients, especially in the context of liver surgery, to avoid elevated mortality. Of interest, liver cirrhosis itself had no impact on survival in our cohort nor in the subgroups (Supplemental Fig. 1A-C), indicating that combination of impaired immune system due to cirrhosis and additional acute infection might be a necessity for our findings.

Moreover, we identified in patents with HCC, that non-anatomical tumor resection and therefor tissue sparing surgery had a positive effect on overall survival. Interestingly extend of surgery had no effect on OS in patients with CCA. This might again be related to the higher number of patients with preexisting liver cirrhosis in the group of HCC patients, although as stated before cirrhosis had no independent influence on OS in our cohort. However, it should be highlighted that all patients were carefully screened before surgery and it was ensured that the remaining liver function after surgery was sufficient. This probably explains why liver function based on the CHILD stage alone was not a decisive factor in our cohort. It should also be noted that extended liver surgery is often associated with larger or less favorably located liver tumors, which could also explain the difference in survival in HCC. In general, after surgery on a cirrhotic liver, at least about 40% of the liver tissue needs to be preserved [18] and our data support non-anatomical- parenchyma sparing- resections at least in HCC patients if possible [19–22].

Furthermore, our data suggest a better OS for patients with chronic viral hepatitis B or C, independent of perioperative infection. Previous studies demonstrated a comparable OS for viral and ALD triggered HCC [23]. However, in our cohort an influence of the ongoing viral infection and potential concomitant immunological effects on OS is possible. Chronic viral infections are a common cause of cirrhosis and HCC. The immune response following viral infection may have a relevant impact on liver-derived tumors, which has been extensively studied in HCC [24]. Interestingly, this observation matches data from several recent treatment studies for liver tumors, which also indicated better treatment success in patients with underlying viral hepatitis B or C [25]. Whether the improved survival of patients with viral hepatitis in our cohort is also related to immunological processes or due to other confounders, cannot be conclusively clarified with the present data. However, the immunological differences between viral and non-viral HCC demonstrate the relevant influence of the immune system on the tumor disease and thus survival in a postoperative setting.

The data presented here highlight the role of perioperative infections as a important parameter in patients with HCC (but not with CCA) for at least short term survival and further endorse the role of liver cirrhosis as a risk factor in patients receiving (major) liver surgery. Furthermore, tissue sparing liver resection in patients with HCC seem to improve long term survival. Nevertheless, our analyses face important limitations, which are due to the study design and cannot be avoided. First, our study included only 202 patients, representing a limited cohort of patients when analyzing complex endpoints such as overall survival. Second, our study represents a retrospective analysis conducted at a single center only, thus center-specific bias cannot be excluded. Due to the retrospective format data collection is often incomplete and important potential confounders as severity of liver disease, comorbidities, and perioperative care practices can’t be always reflected in detail. Additionally, patients were included over a long period of time during which the assessment of infections as well as operation techniques might have changed. Especially perioperative management and infection prevention has undergone changes over this long period of time, generally infection control is supposed to have improved, but multi-resistant bacteria are emerging as well and complication treatment. Moreover, postoperative increase in markers for inflammation like CRP is common as well as detection of bacteria in intraoperative bile samples. Therefor misclassification of “infection” is a possible confounder, even by using only a combination of increased inflammation markers and pathogen detection for defining “infection”. In addition, patients with two different tumor entities were included. Furthermore, our study cannot distinguish whether it is really the extent of the surgical resection or the size of the liver tumor that necessitated the extensive resection that is responsible for the differences in overall survival. Finally, our data does not provide evidence on whether an individual patient might have benefitted similarly or even more in terms of long-term survival, from a different treatment modality such as systemic treatment or other locally ablative therapies. This important clinical question can only be answered by further prospective clinical studies including different treatment modalities and providing a streamlined treatment protocol avoiding bias due to incomplete data collection and treatment changes over time as stated before. Such studies would not only improve the clinical management of patients with liver cancer but could also provide important insights into the pathophysiology of HCC (and CCA) if appropriate translational programs are integrated into the respective study-design. We hope that in the long term this study will help to improve the perioperative management of patients with liver tumors and encourage those needed further prospective studies, particularly to improve infection control in this patient group.

## Conclusion

In summary the data presented here underscores the impact of perioperative infections as one potential complication effecting the overall survival and especially short-term survival of patients undergoing surgery for primary liver tumors. Most importantly, this effect was only observed in patients with HCC, but not with CCA, impressively demonstrating the differences in the pathophysiology of the two diseases and the different patient groups affected. In line with this, also the extension of liver resection only had impact on OS in patients with HCC, but not CCA.

## Supplementary Information

Supplementary Material 1.

Supplementary Material 2.

## Data Availability

No datasets were generated or analysed during the current study.
