# Supplementary figures and images for: Perioperative infections as a prognostic risk factor in hepatocellular carcinoma and cholangiocellular carcinoma: a comparative analysis
Source: World J Surg Oncol. 2025 Jan 7;23:9. doi: 10.1186/s12957-024-03651-8 (PMC11705658; doi:10.1186/s12957-024-03651-8)

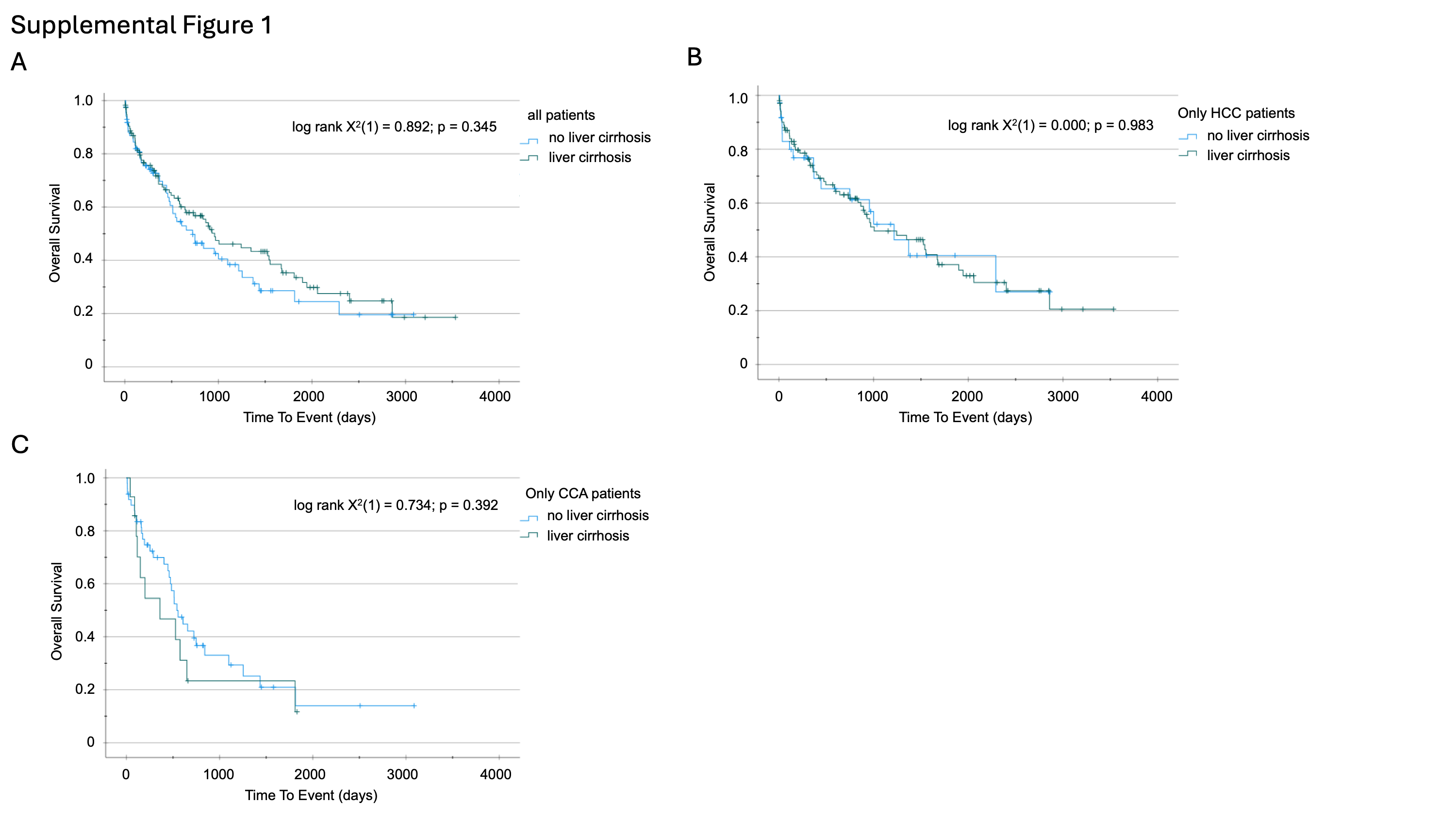

Supplement: Supplementary file 1 — Supplementary Material 1. [file 12957_2024_3651_MOESM1_ESM.png]
