# Supplementary material for: Perioperative infections as a prognostic risk factor in hepatocellular carcinoma and cholangiocellular carcinoma: a comparative analysis
Source: World J Surg Oncol. 2025 Jan 7;23:9. doi: 10.1186/s12957-024-03651-8 (PMC11705658; doi:10.1186/s12957-024-03651-8)
Supplement: Supplementary file 2 — Supplementary Material 2. [file 12957_2024_3651_MOESM2_ESM.docx]

**Supplemental Table 1: Univariate Cox regression analysis for OS in patients operated on HCC**

| **Parameter** | **p-value** | **Hazard Ratio (95% CI)** | **n (139)** |
| --- | --- | --- | --- |
| Age | 0.572 | 1.007 (0.983-1.032) | 139 |
| Sex | 0.812 | 0.931 (0.516-1.681) | 139 |
| Weight | 0.459 | 0.995 (0.982-1.008) | 133 |
| Hight | 0.388 | 1.011 (0.986-1.037) | 133 |
| BMI | 0.152 | 0.963 (0.915-1.014) | 133 |
| Infection | 0.003 | 2.024 (1.273-3.217) | 139 |
| Active viral Hepatitis | 0.097 | 0.659 (0.403-1.078) | 138 |
| Non-anatomical (limited) liver resection | 0.022 | 0.567 (0.349-0.921) | 139 |
| Sodium | 0.057 | 0.915 (0.836-1.003) | 136 |
| Potassium | 0.770 | 1.095 (0.595-2.016) | 135 |
| Calcium | 0.024 | 0.137 (0.024-0.770) | 134 |
| Creatinin | 0.238 | 1.528 (0.756-3.090) | 136 |
| GFR | 0.639 | 0.998 (0.987-1.008) | 135 |
| Urea | 0.385 | 1.006 (0.992-1.020) | 135 |
| Uric acid | 0.221 | 1.136 (0.926-1.395) | 40 |
| Bilirubin | <0.001 | 1.489 (1.178-1.881) | 126 |
| CRP | <0.001 | 1.273 (1.174-1.381) | 135 |
| LDH | 0.129 | 1.001 (1.000-1.002) | 55 |
| AST | 0.012 | 1.004 (1.001-1.007) | 135 |
| γGT | <0.001 | 1.001 (1.001-1.002) | 135 |
| AP | <0.001 | 1.003 (1.002-1.005) | 102 |
| Albumin | 0.705 | 0.992 (0.950-1.035) | 33 |
| TSH | 0.183 | 1.123 (0.947-1.332) | 108 |
| leucocyte count | 0.133 | 1.087 (0.975-1.213) | 135 |
| Hb | 0.001 | 0.818 (0.726-0.922) | 136 |
| Thrombocytes | 0.783 | 1.000 (0.997-1.002) | 136 |
| CEA | 0.286 | 0.729 (0.407-1.304) | 18 |
| AFP | 0.026 | 1.000 (1.000-1.000) | 26 |
| CA-19-9 | 0.605 | 1.002 (0.995-1.008) | 20 |
| INR | 0.032 | 8.915 (1.209-65.746) | 134 |
| PTT | <0.001 | 1.030 (1.012-1.048) | 134 |

BMI: Body-Mass Index, GFR: glomerular filtration rate, CRP: C-reactive protein; LDH: lactate dehydrogenase; AST: aspartate-aminotransferase, γGT: γ-glutamyltransferase; AP: Alkaline phosphatase; TSH: Thyroid-stimulating hormone; Hb: Hemoglobin; CEA: Carcinoembryonic antigen, AFP: α-fetoprotein, CA19-9: Carbohydrate antigen 19-9, INR: International normalized ratio, aPTT: activated partial thromboplastin time

**Supplemental Table 2: Univariate Cox regression analysis for OS in patients operated on CCA**

| **Parameter** | **p-value** | **Hazard Ratio (95% CI)** | **n (139)** |
| --- | --- | --- | --- |
| Age | 0.078 | 1.028 (0.997-1.061) | 63 |
| Sex | 0.547 | 1.205 (0.657-2.207) | 63 |
| Weight | 0.165 | 0.988 (0.971-1.005) | 63 |
| Hight | 0.164 | 0.975 (0.941-1.010) | 63 |
| BMI | 0.331 | 0.968 (0.908-1.033) | 63 |
| Infection | 0.517 | 1.223 (0.665-2.249) | 63 |
| Active viral Hepatitis | 0.857 | 0.917 (0359.-2.343) | 62 |
| Non-anatomical (limited) liver resection | 0.525 | 1.226 (0.654-2.300) | 63 |
| Sodium | 0.895 | 0.995 (0.921-1.075) | 62 |
| Potassium | 0.984 | 1.001 (0.953-1.051) | 62 |
| Calcium | 0.040 | 0.243 (0.063-0.937) | 61 |
| Creatinin | 0.655 | 0.937 (0.704-1.246) | 62 |
| GFR | 0.987 | 1.000 (0.988-1.012) | 61 |
| Urea | 0.410 | 1.009 (0.988-1.029) | 62 |
| Uric acid | 0.742 | 0.992 (0.947-1.039) | 23 |
| Bilirubin | 0.486 | 1.022 (0.961-1.088) | 57 |
| CRP | 0.025 | 1.093 (1.011-1.180) | 61 |
| LDH | 0.119 | 1.001 (1.000-1.002) | 31 |
| AST | 0.795 | 1.001 (0.995-1.007) | 61 |
| γGT | 0.297 | 1.001 (0.999-1.002) | 58 |
| AP | 0.342 | 1.001 (0.999-1.002) | 47 |
| Albumin | 0.409 | 1.019 (0.974-1.067) | 18 |
| TSH | 0.912 | 1.010 (0.850-1.200) | 50 |
| leucocyte count | 0.262 | 1.056 (0.960-1.160) | 62 |
| Hb | 0.964 | 1.000 (0.989-1.012) | 62 |
| Thrombocytes | 0.566 | 1.001 (0.998-1.003) | 62 |
| CEA | 0.071 | 1.071 (0.994-1.154) | 33 |
| AFP | 0.931 | 1.002 (0.968-1.037) | 39 |
| CA-19-9 | 0.065 | 1.000 (1.000-1.000) | 40 |
| INR | 0.739 | 1.514 (0.132-17.330) | 51 |
| PTT | 0.026 | 1.063 (1.007-1.123) | 51 |

BMI: Body-Mass Index, GFR: glomerular filtration rate, CRP: C-reactive protein; LDH: lactate dehydrogenase; AST: aspartate-aminotransferase, γGT: γ-glutamyltransferase; AP: Alkaline phosphatase; TSH: Thyroid-stimulating hormone; Hb: Hemoglobin; CEA: Carcinoembryonic antigen, AFP: α-fetoprotein, CA19-9: Carbohydrate antigen 19-9, INR: International normalized ratio, aPTT: activated partial thromboplastin time
